# Supplementary material for: XAC4296 Is a Multifunctional and Exclusive Xanthomonadaceae Gene Containing a Fusion of Lytic Transglycosylase and Epimerase Domains
Source: Microorganisms. 2022 May 11;10(5):1008. doi: 10.3390/microorganisms10051008 (PMC9143381; doi:10.3390/microorganisms10051008)
Supplement: Supplementary file 1 [file microorganisms-10-01008-s001.zip › Figure S2.pdf]

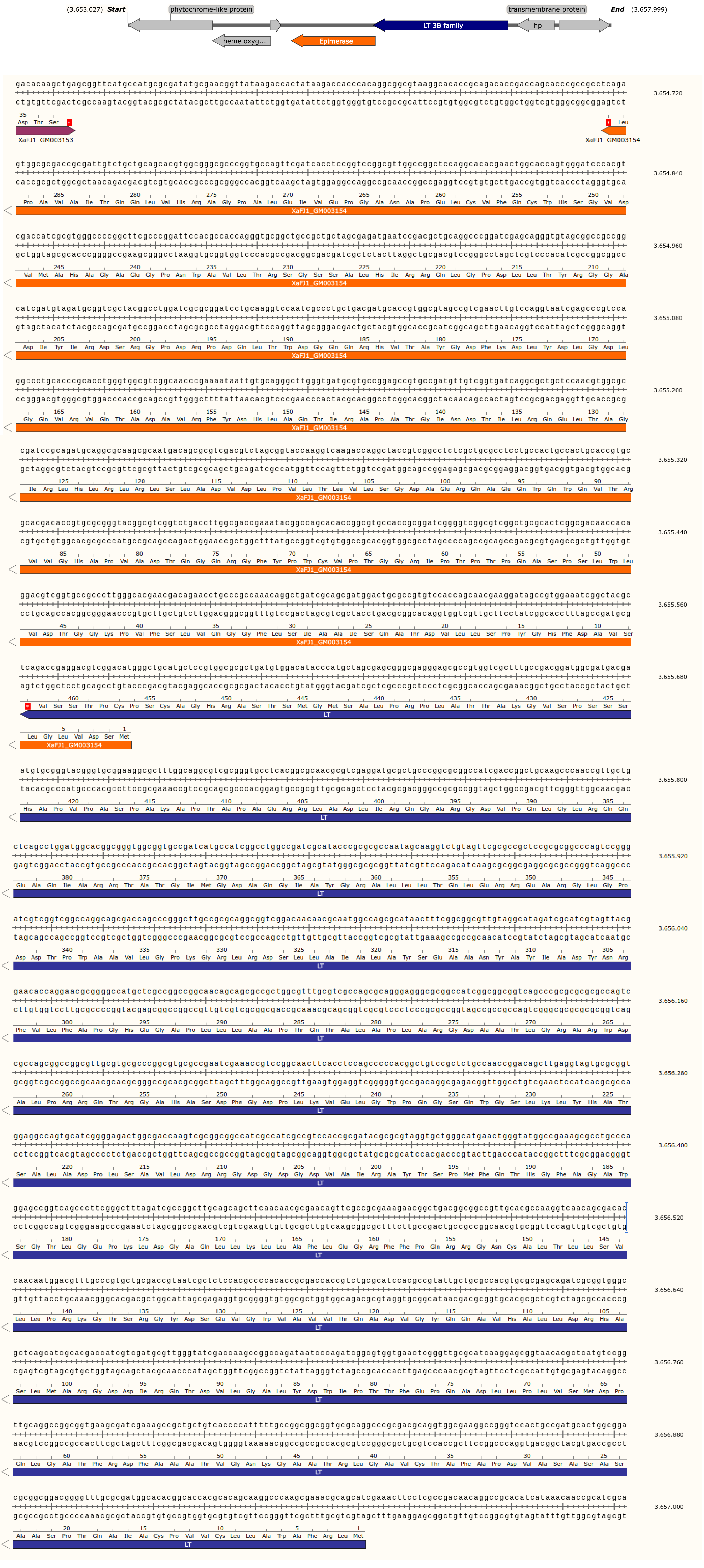

(3.653.027) Start
phytochrome-like protein
transmembrane protein
End (3.657.999)
LT 3B family
hp
heme oxyg...
Epimerase
XaFJ1_GM003153
XaFJ1_GM003154
LT
3.654.720
3.654.840
3.654.960
3.655.080
3.655.200
3.655.320
3.655.440
3.655.560
3.655.680
3.655.800
3.655.920
3.656.040
3.656.160
3.656.280
3.656.400
3.656.520
3.656.640
3.656.760
3.656.880
3.657.000
