# Supplementary material for: XAC4296 Is a Multifunctional and Exclusive Xanthomonadaceae Gene Containing a Fusion of Lytic Transglycosylase and Epimerase Domains
Source: Microorganisms. 2022 May 11;10(5):1008. doi: 10.3390/microorganisms10051008 (PMC9143381; doi:10.3390/microorganisms10051008)
Supplement: Supplementary file 1 [file microorganisms-10-01008-s001.zip › Figure S4.pdf]

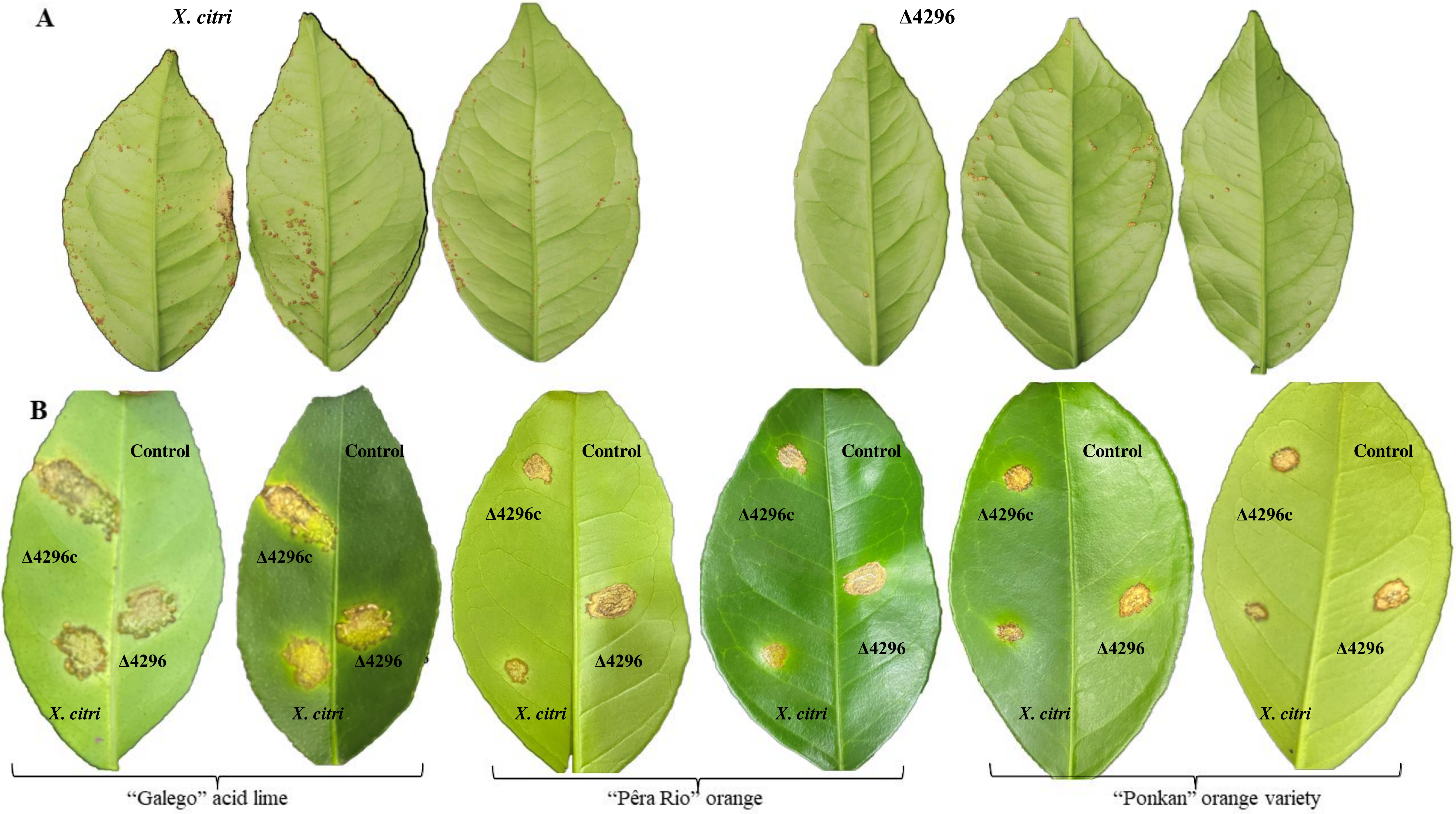
A
X. citri
Δ4296
B
Control
Δ4296c
Δ4296
X. citri
Control
Δ4296c
Δ4296
X. citri
Control
Δ4296c
Δ4296
X. citri
Control
Δ4296c
Δ4296
X. citri
Control
Δ4296c
Δ4296
X. citri
Control
Δ4296c
Δ4296
X. citri
"Galego" acid lime
"Pêra Rio" orange
"Ponkan" orange variety
