# Supplementary material for: XAC4296 Is a Multifunctional and Exclusive Xanthomonadaceae Gene Containing a Fusion of Lytic Transglycosylase and Epimerase Domains
Source: Microorganisms. 2022 May 11;10(5):1008. doi: 10.3390/microorganisms10051008 (PMC9143381; doi:10.3390/microorganisms10051008)
Supplement: Supplementary file 1 [file microorganisms-10-01008-s001.zip › Table S1.pdf]

Table S1. Xanthomonadaceae used for comparative genomics. In this table, there are 2 species for the *Pseudoxanthomonas* genus, 36 species for the *Stenotrophomonas* genus, and 270 species for the *Xanthomonas* genus.

Genus	Species	Accession number
Pseudoxanthomonas	*Pseudoxanthomonas sp. isolate* SSD1	CP059266.1
	Pseudoxanthomonas suwonensis strain J1	CP011144.1
Stenotrophomonas	*Stenotrophomonas maltophilia* ZT1	CP019797.1
	Stenotrophomonas acidaminiphila strain T0-18	CP043567.1
	Stenotrophomonas acidaminiphila strain T25-65	CP043570.1
	Stenotrophomonas acidaminiphila strain ZAC14D2_NAIMI4_2	CP012900.1
	Stenotrophomonas indicatrix strain DAIF1	CP037883.1
	Stenotrophomonas maltophilia strain AA1	CP018756.1
	Stenotrophomonas maltophilia strain MER1	CP049368.1
	Stenotrophomonas maltophilia strain PEG-305	CP040437.1
	Stenotrophomonas maltophilia strain PEG-390	CP040436.1
	Stenotrophomonas maltophilia strain CPBW01	CP047310.1
	Stenotrophomonas maltophilia strain ISMMS2R	CP011306.1
	Stenotrophomonas maltophilia strain ISMMS2	CP011305.1
	Stenotrophomonas sp. MYb57	CP023271.1
	Stenotrophomonas sp. NA06056	CP054931.1
	Stenotrophomonas sp. SAU14A_NAIMI4_5	CP026003.1
	Stenotrophomonas sp. 364	CP047135.1
	Stenotrophomonas sp. CW117	CP062156.1
	Stenotrophomonas sp. ESTM1D_MKCIP4_1	CP026004.1
	Stenotrophomonas maltophilia strain U5	CP040429.1
	Stenotrophomonas nitritireducens strain 2001	CP016756.1
	Stenotrophomonas rhizophila strain DSM14405	CP007597.1
	Stenotrophomonas rhizophila strain JC1	CP050062.1
	Stenotrophomonas rhizophila strain QL-P4	CP016294.1
	Stenotrophomonas sp. 169	CP061204.1
	Stenotrophomonas sp. G4	CP031741.1
	Stenotrophomonas sp. KCTC 12332 strain YM1	CP014274.1
	Stenotrophomonas sp. LM091	CP017483.1
	Stenotrophomonas sp. SAU14A_NAIMI4_8	CP025999.1
	Stenotrophomonas sp. SXG-1	CP046588.1

	Stenotrophomonas sp. YAU14A_MKIMI4_1	CP025998.1
	Stenotrophomonas sp. YAU14D1_LEIMI4_1	CP025997.1
	Stenotrophomonas sp. ZAC14A_NAIMI4_1	CP026002.1
	Stenotrophomonas sp. ZAC14D1_NAIMI4_1	CP026001.1
	Stenotrophomonas sp. ZAC14D1_NAIMI4_6	CP026000.1
	Stenotrophomonas sp. ZAC14D2_NAIMI4_6	CP025996.1
	Stenotrophomonas sp. ZAC14D2_NAIMI4_7	CP025995.1
Xanthomonas	*Xanthomonas arboricola* pv. *juglandis* isolate 3	LR861807.1
	Xanthomonas arboricola pv. *juglandis* strain Xaj 417	CP012251.1
	Xanthomonas arboricola pv. pruni strain 15-088	CP044334.1
	Xanthomonas arboricola strain 17	CP011256.1
	Xanthomonas axonopodis Xac29-1	CP004399.1
	Xanthomonas axonopodis pv. *citri* str. 306	AE008923.1
	Xanthomonas axonopodis pv. *citrumelo* F1	CP002914.1
	Xanthomonas axonopodis pv. *commiphoreae* strain LMG26789	CP031059.1
	Xanthomonas phaseoli pv. *dieffenbachiae* LMG 695	CP014347.1
	Xanthomonas axonopodis pv. *phaseoli* strain CFBP412	CP020964.1
	Xanthomonas citri pv. *malvacearum* strain HD-1	CP046019.1
	Xanthomonas citri pv. *malvacearum* strain MS14003	CP023159.1
	Xanthomonas citri pv. *malvacearum* strain MSCT	CP017020.1
	Xanthomonas citri pv. *malvacearum* strain XcmH1005	CP013004.1
	Xanthomonas citri pv. *malvacearum* strain XcmN1003	CP013006.1
	Xanthomonas citri pv. *phaseoli* var. *fuscans* strain CFBP4885	CP020992.1
	Xanthomonas citri pv. *phaseoli* var. *fuscans* strain CFBP6165	CP020998.1
	Xanthomonas citri pv. *phaseoli* var. *fuscans* strain CFBP6166	CP021001.1
	Xanthomonas citri pv. *phaseoli* var. *fuscans* strain CFBP6167	CP021018.1
	Xanthomonas citri pv. *phaseoli* var. *fuscans* strain CFBP6975	CP021006.1
	Xanthomonas oryzae pv. *oryzae* PXO86	CP007166.1
	Xanthomonas oryzae pv. *oryzae* PXO99A	CP000967.2
	Xanthomonas oryzae pv. *oryzae* strain AUST2013	CP033196.1
	Xanthomonas oryzae pv. *oryzae* strain AXO1947	CP013666.1
	Xanthomonas oryzae pv. *oryzae* strain BAI3	CP025610.1
	Xanthomonas oryzae pv. *oryzae* strain BXO1	CP033201.1

	Xanthomonas oryzae pv. *oryzae* strain BXO512	CP033185.1<br>CP065228.1
	Xanthomonas oryzae pv. *oryzae* strain CFBP1948	
	Xanthomonas oryzae pv. *oryzae* strain CFBP1949	CP033184.1
	Xanthomonas oryzae pv. *oryzae* strain CFBP1951	CP033183.1
	Xanthomonas oryzae pv. *oryzae* strain Ug11	CP033170.1
	Xanthomonas oryzae pv. *oryzae* strain XF89b	CP011532.1
	Xanthomonas oryzae pv. *oryzae* strain K3a	CP050115.1
	Xanthomonas oryzae pv. *oryzae* strain K3	CP050114.1
	Xanthomonas oryzae pv. *oryzae* strain KXO85	CP033197.1
	Xanthomonas oryzae pv. *oryzae* strain MAI1	CP025609.1
	Xanthomonas oryzae pv. *oryzae* strain MAI68	CP019085.1
	Xanthomonas oryzae pv. *oryzae* strain MAI73	CP019086.1
	Xanthomonas oryzae pv. *oryzae* strain MAI95	CP019087.1
	Xanthomonas oryzae pv. *oryzae* strain MAI99	CP019088.1
	Xanthomonas oryzae pv. *oryzae* strain XM9	CP020334.1
	Xanthomonas oryzae pv. *oryzae* strain YC11	CP031464.1
	Xanthomonas oryzae pv. *oryzae* strain YN24	CP018089.1
	Xanthomonas oryzae pv. *oryzicola* BLS256	CP003057.2
	Xanthomonas oryzae pv. *oryzicola* strain 0-9	CP045912.1
	Xanthomonas oryzae pv. *oryzicola* strain B8-12	CP011955.1
	Xanthomonas oryzae pv. *oryzicola* strain BLS279	CP011956.1
	Xanthomonas oryzae pv. *oryzicola* strain BXOR1	CP011957.1
	Xanthomonas oryzae pv. *oryzicola* strain CFBP2286	CP011962.1
	Xanthomonas oryzae pv. *oryzicola* strain CFBP7331	CP011958.1
	Xanthomonas translucens pv. *undulosa* strain Xtu 4699	CP008714.1
	Xanthomonas vasicola pv. *arecae* strain NCPPB 2649	CP034653.1
	Xanthomonas vasicola pv. *vasculorum* strain SAM119	CP028127.1
	Xanthomonas vasicola pv. *vasculorum* strain Xv1601	CP025272.1
	Xanthomonas vasicola strain NCPPB 902	CP034657.1
	Xanthomonas vasicola strain NCPPB 1060	CP034649.1
	Xanthomonas vesicatoria ATCC 35937 strain LMG911	CP018725.1
	Xanthomonas vesicatoria strain LM159	CP018470.1
	Xanthomonas hortorum pv. *vitians* LM16734	CP060399.1
	Xanthomonas hortorum pv. vitians strain CFBP 498	LR828257.1
	Xanthomonas axonopodis pv. *phaseoli* strain CFBP6164	CP020967.1
	Xanthomonas axonopodis pv. *phaseoli* strain CFBP6546R	CP020971.1
	Xanthomonas axonopodis pv. *phaseoli* strain	CP020975.1

	CFBP6982	
	Xanthomonas axonopodis pv. *phaseoli* strain ISO18C2	CP012048.1
	Xanthomonas axonopodis pv. *phaseoli* strain ISO18C8	CP012063.1
	Xanthomonas phaseoli pv. *phaseoli* strain ISO98C12	CP012057.1
	Xanthomonas axonopodis pv. *vasculorum* strain NCPPB 796	CP053649.1
	Xanthomonas campestris pv. badrii strain NEB122	CP051651.1
	Xanthomonas campestris pv. *campestris* MAFF106712	AP019682.1
	Xanthomonas campestris pv. *campestris* MAFF302021	AP019684.1
	Xanthomonas campestris pv. *campestris* complete genome, strain B100	AM920689.1
	Xanthomonas campestris pv. *campestris* strain 17	CP011946.1
	Xanthomonas campestris pv. *campestris* strain 3811	CP025750.1
	Xanthomonas campestris pv. *campestris* strain ICMP 4013	CP012146.1
	Xanthomonas campestris pv. *campestris* strain ICMP 21080	CP012145.1
	Xanthomonas campestris pv. *campestris* strain Xcc8004_Xcc1	CP029483.1
	Xanthomonas campestris pv. *campestris* strain Xcc8004_Xcc2	CP029484.1
	Xanthomonas citri pv. *phaseoli* var. *fuscans* strain CFBP6988R	CP020979.1
	Xanthomonas citri pv. *phaseoli* var. *fuscans* strain CFBP6989 CP020981.1	CP020981.1
	Xanthomonas citri pv. *phaseoli* var. *fuscans* strain CFBP6990	CP020983.1
	Xanthomonas citri pv. *phaseoli* var. *fuscans* strain CFBP6991	CP021015.1
	Xanthomonas citri pv. *phaseoli* var. *fuscans* strain CFBP6992	CP020985.1
	Xanthomonas citri pv. *phaseoli* var. *fuscans* strain CFBP6994R	CP020987.1
	Xanthomonas citri pv. *phaseoli* var. *fuscans* strain CFBP6996R	CP020989.1
	Xanthomonas citri pv. *punicae* strain BD0022	CP030159.1
	Xanthomonas citri pv. *punicae* strain BD0023	CP030161.1
	Xanthomonas citri pv. *punicae* strain BD0025	CP030169.1
	Xanthomonas citri pv. *punicae* strain LMG7439	CP030164.1
	Xanthomonas citri pv. *punicae* strain LMG7504	CP030166.1
	Xanthomonas citri pv. *punicae* strain LMG 859	CP030178.1
	Xanthomonas citri pv. *punicae* strain NCPPB 3563	CP030163.1
	Xanthomonas citri pv. *vignicola* strain CFBP7111	CP022263.1
	Xanthomonas citri pv. *vignicola* strain CFBP7112	CP022267.1
	Xanthomonas citri pv. *vignicola* strain CFBP7113	CP022270.1
	Xanthomonas oryzae pv. *oryzae* strain CFBP1952	CP033182.1

	Xanthomonas oryzae pv. *oryzae* strain CFBP7319	CP033181.1
	Xanthomonas oryzae pv. *oryzae* strain CFBP7320	CP033186.1
	Xanthomonas oryzae pv. *oryzae* strain CFBP7321	CP033180.1
	Xanthomonas oryzae pv. *oryzae* strain CFBP7322	CP033179.1
	Xanthomonas oryzae pv. *oryzae* strain CFBP7323	CP033178.1
	Xanthomonas oryzae pv. *oryzae* strain CFBP7324	CP033177.1
	Xanthomonas oryzae pv. *oryzae* strain CFBP7325	CP033176.1
	Xanthomonas oryzae pv. *oryzae* strain CFBP7337	CP033175.1
	Xanthomonas oryzae pv. *oryzae* strain CFBP7340	CP033174.1
	Xanthomonas oryzae pv. *oryzae* strain CFBP8172	CP033173.1
	Xanthomonas oryzae pv. *oryzae* strain CIAT	CP033194.1
	Xanthomonas oryzae pv. *oryzae* strain CIX298	CP036378.1
	Xanthomonas oryzae pv. *oryzae* strain CIX2374	CP036377.1
	Xanthomonas oryzae pv. *oryzae* strain Dak16	CP033172.1
	Xanthomonas oryzae pv. *oryzae* strain HuN37	CP031456.1
	Xanthomonas oryzae pv. *oryzae* strain ICMP3125	CP031697.1
	Xanthomonas oryzae pv. *oryzae* strain MAI106	CP019089.1
	Xanthomonas oryzae pv. *oryzae* strain MAI129	CP019090.1
	Xanthomonas oryzae pv. *oryzae* strain MAI134	CP019091.1
	Xanthomonas oryzae pv. *oryzae* strain MAI145	CP019092.1
	Xanthomonas oryzae pv. *oryzae* strain NX0260	CP033192.1
	Xanthomonas oryzae pv. *oryzae* strain OS198	CP031461.1
	Xanthomonas oryzae pv. *oryzae* strain PX079	CP031462.1
	Xanthomonas oryzae pv. *oryzae* strain PX086	CP031463.1
	Xanthomonas oryzae pv. *oryzae* strain PXO61	CP020942.1
	Xanthomonas oryzae pv. *oryzae* strain PXO61	CP021788.1
	Xanthomonas oryzae pv. *oryzae* strain PXO61	CP021789.1
	Xanthomonas oryzae pv. *oryzae* strain PXO61	CP033187.3
	Xanthomonas oryzae pv. *oryzae* strain PXO71	CP013670.1
	Xanthomonas oryzae pv. *oryzae* strain PXO83	CP012947.1
	Xanthomonas oryzae pv. *oryzae* strain PXO142	CP031698.1
	Xanthomonas oryzae pv. *oryzae* strain PXO145	CP013961.1
	Xanthomonas oryzae pv. *oryzae* strain PXO211	CP013674.1
	Xanthomonas oryzae pv. *oryzicola* strain CFBP7341	CP011959.1
	Xanthomonas oryzae pv. *oryzicola* strain CFBP7342	CP007221.1
	Xanthomonas oryzae pv. *oryzicola* strain GX01	CP043403.1
	Xanthomonas oryzae pv. *oryzicola* strain L8	CP011960.1
	Xanthomonas oryzae pv. *oryzicola* strain RS105	CP011961.1
	Xanthomonas oryzae pv. *oryzicola* strain YM15	CP007810.1
	Xanthomonas oryzae strain BAI23	CP036256.1
	Xanthomonas oryzae strain BB151-3	CP036255.1
	Xanthomonas oryzae strain BB156-2	CP036254.1
	Xanthomonas oryzae strain NCPPB 4346	CP036253.1
	Xanthomonas oryzae strain NJ611	CP036252.1
	Xanthomonas oryzae strain X11-5A	CP036251.1
	Xanthomonas perforans 91-118	CP019725.1
	Xanthomonas perforans strain LH3	CP018475.1

	Xanthomonas sacchari strain R1	CP010409.1
	Xanthomonas sp. CPBF 367 isolate 1	LR861803.1
	Xanthomonas sp. CPBF 426	LR861805.1
	Xanthomonas hortorum strain B07-007	CP016878.1
	Xanthomonas hortorum strain VT106	CP031607.1
	Xanthomonas hyacinthi strain CFBP 1156	CP043476.1
	Xanthomonas oryzae pv. *oryzae* KACC 10331	AE013598.1
	Xanthomonas oryzae pv. *oryzae* MAFF 311018	AP008229.1
	Xanthomonas oryzae pv. *oryzae* strain PXO524	CP013677.1
	Xanthomonas oryzae pv. *oryzae* strain PXO563	CP013678.1
	Xanthomonas oryzae pv. *oryzae* strain PXO602	CP013679.1
	Xanthomonas oryzae pv. *oryzae* strain SK2-3	CP019515.1
	Xanthomonas oryzae pv. *oryzae* strain ScYc-b	CP018087.1
	Xanthomonas oryzae pv. *oryzae* strain ScYc-b	CP031469.1
	Xanthomonas oryzae pv. *oryzae* strain T19	CP033171.1
	Xanthomonas oryzae pv. *oryzae* strain PXO364	CP033191.1
	Xanthomonas oryzae pv. *oryzae* strain PXO404	CP033190.1
	Xanthomonas oryzae pv. *oryzae* strain PXO421	CP033189.1
	Xanthomonas oryzae pv. *oryzae* strain PXO513	CP033188.1
	Xanthomonas citri pv. *malvacearum* strain AR81009	CP023155.1
	Xanthomonas campestris pv. *campestris* str. ATCC 33913	AE008922.1
	Xanthomonas campestris pv. *campestris* str. CN03	CP017308.1
	Xanthomonas campestris pv. *campestris* str. CN12	CP017310.1
	Xanthomonas campestris pv. *campestris* str. CN14	CP017317.1
	Xanthomonas campestris pv. *campestris* str. CN15	CP017323.1
	Xanthomonas campestris pv. *campestris* str. CN16	CP017389.1
	Xanthomonas campestris pv. *campestris* str. CN17	CP017307.1
	Xanthomonas campestris pv. *campestris* str. CN18	CP017319.1
	Xanthomonas campestris pv. *musacearum* NCPPB 4379	CP034655.1
	Xanthomonas campestris pv. *raphani* 756C	CP002789.1
	Xanthomonas campestris pv. *raphani* strain MAFF106181	CP058243.1
	Xanthomonas campestris pv. *vesicatoria*	AM039952.1
	Xanthomonas campestris pv. *vesicatoria* str. 85-10	CP017190.1
	Xanthomonas campestris strain M28	CP062066.1
	Xanthomonas citri pv. *anacardii* CFBP 2913	CP024057.1
	Xanthomonas citri pv. *citri* strain 03-1638-1-1	CP023285.1
	Xanthomonas citri pv. *citri* strain DAR72029	CP060446.1
	Xanthomonas citri pv. *citri* strain DAR73886	CP060465.1
	Xanthomonas citri strain UnB-Xtec2D	CP048044.1
	Xanthomonas citri strain UnB-XtecTG02-2	CP048712.1
	Xanthomonas citri subsp. *citri* A306	CP006857.1
	Xanthomonas citri subsp. *citri* Aw12879	CP003778.1
	Xanthomonas citri subsp. *citri* UI6	CP008992.1
	Xanthomonas citri subsp. *citri* strain 5208	CP009028.1
	Xanthomonas citri subsp. *citri* strain AW13	CP009031.1

	Xanthomonas citri subsp. *citri* strain AW14	CP009034.1
	Xanthomonas citri subsp. *citri* strain AW15	CP009037.1
	Xanthomonas citri subsp. *citri* strain AW16	CP009040.1
	Xanthomonas citri subsp. *citri* strain BL18	CP009025.1
	Xanthomonas citri subsp. *citri* strain FB19	CP009022.1
	Xanthomonas citri subsp. *citri* strain MN10	CP009004.1
	Xanthomonas citri subsp. *citri* strain MN11	CP009001.1
	Xanthomonas citri subsp. *citri* strain MN12	CP008998.1
	Xanthomonas citri subsp. *citri* strain NT17	CP008995.1
	Xanthomonas citri subsp. *citri* strain UI7	CP008989.1
	Xanthomonas citri subsp. *citri* strain gd2	CP009019.1
	Xanthomonas oryzae pv. *oryzae* strain ITCCBB0002	CP046148.1
	Xanthomonas oryzae pv. *oryzae* strain IXO704	CP040604.1
	Xanthomonas oryzae pv. *oryzae* strain IXO1088	CP040687.1
	Xanthomonas oryzae pv. *oryzae* strain IX-280	CP019226.1
	Xanthomonas oryzae pv. *oryzae* strain JL25	CP031457.1
	Xanthomonas oryzae pv. *oryzae* strain JL28	CP031458.1
	Xanthomonas oryzae pv. *oryzae* strain JL33	CP031459.1
	Xanthomonas oryzae pv. *oryzae* strain JP01	CP031460.1
	Xanthomonas oryzae pv. *oryzae* strain JW11089	CP033193.2
	Xanthomonas oryzae pv. *oryzae* strain K1	CP049205.1
	Xanthomonas oryzae pv. *oryzae* strain K2	CP050113.1
	Xanthomonas citri subsp. *citri* strain gd3	CP009016.1
	Xanthomonas citri subsp. *citri* strain jx4	CP009013.1
	Xanthomonas citri subsp. *citri* strain jx5	CP009010.1
	Xanthomonas citri pv. *citri* strain DAR73889	CP060470.1
	Xanthomonas citri pv. *citri* strain DAR73910	CP060476.1
	Xanthomonas citri pv. *citri* strain DAR84832	CP060460.1
	Xanthomonas citri pv. *glycines* strain 1018	CP041961.1
	Xanthomonas oryzae pv. *oryzae* strain PXO236	CP013675.1
	Xanthomonas oryzae pv. *oryzae* strain PXO282	CP013676.1
	Xanthomonas hortorum pv. *gardneri* strain JS749-3	CP018728.1
	Xanthomonas hortorum pv. *pelargonii* strain CFBP 2533	LR828261.1
	Xanthomonas citri pv. *citri* strain LH201	CP018858.1
	Xanthomonas citri pv. *citri* strain LH276	CP018854.1
	Xanthomonas citri pv. *citri* strain LJ207-7	CP018850.1
	Xanthomonas citri pv. *citri* strain LL074-4	CP018847.1
	Xanthomonas citri pv. *citri* strain TX160042	CP020882.1
	Xanthomonas citri pv. *citri* strain TX160149	CP020885.1
	Xanthomonas citri pv. *citri* strain TX160197	CP020889.1
	Xanthomonas citri pv. *citri* strain Xcc29-1	CP023661.1
	Xanthomonas citri pv. *citri* strain Xcc49	CP023662.1
	Xanthomonas citri pv. *citri* strain jx-6	CP011827.2
	Xanthomonas citri pv. *fuscans* CFBP 6988	CP026331.1
	Xanthomonas citri pv. *fuscans* strain Xff49	CP023294.1
	Xanthomonas citri pv. *glycines* strain 8ra	CP041781.1
	Xanthomonas citri pv. *fuscans* strain ISO118C5	CP012051.1
	Xanthomonas sp. GW	CP051189.1

	Xanthomonas sp. ISO98C4	CP012060.1
	Xanthomonas sp. SI	CP051261.1
	Xanthomonas sp. SS	CP051190.1
	Xanthomonas sp. WG16	CP062255.1
	Xanthomonas translucens pv. *cerealis* strain 01	CP038228.1
	Xanthomonas translucens pv. *translucens* DSM 18974 isolate peng1	LT604072.1
	Xanthomonas translucens pv. undulosa strain ICMP11055	CP009750.1
	Xanthomonas translucens pv. undulosa strain LW16	CP043540.1
	Xanthomonas translucens pv. undulosa strain P3	CP043500.1
	Xanthomonas citri pv. *glycines* strain K2	CP041967.1
	Xanthomonas citri pv. *glycines* str. 8ra	CP017188.2
	Xanthomonas citri pv. *glycines* strain 1157	CP041963.1
	Xanthomonas citri pv. *glycines* str. 12-2	CP015972.1
	Xanthomonas arboricola strain SL2098	CP041965.1
	Xanthomonas citri pv. *glycines* strain EB08	CP026334.1
	Xanthomonas fuscans subsp. *fuscans* strain ISO118C1	CP012053.1
	Xanthomonas fuscans subsp. *fuscans* strain ISO12C3	CP012055.1
	Xanthomonas translucens pv. *undulosa* strain Xtu 4699	CP008714.1
	Xanthomonas vasicola pv. *arecae* strain NCPPB 2649	CP034653.1
	Xanthomonas vasicola pv. *vasculorum* strain SAM119	CP028127.1
	Xanthomonas vasicola pv. *vasculorum* strain Xv1601	CP025272.1
	Xanthomonas vasicola strain NCPPB 902	CP034657.1
	Xanthomonas vasicola strain NCPPB 1060	CP034649.1
	Xanthomonas vesicatoria ATCC 35937 strain LMG911	CP018725.1
	Xanthomonas vesicatoria strain LM159	CP018470.1
	Xanthomonas hortorum pv. *vitians* LM16734	CP060399.1
	Xanthomonas hortorum pv. *vitians* strain CFBP 498	LR828257.1
	Xanthomonas hortorum strain B07-007	CP016878.1
	Xanthomonas hortorum strain VT106	CP031607.1
	Xanthomonas hyacinthi strain CFBP 1156	CP043476.1
	Xanthomonas oryzae pv. *oryzae* KACC 10331	AE013598.1
	Xanthomonas oryzae pv. *oryzae* MAFF 311018 DNA	AP008229.1
	Xanthomonas oryzae pv. *oryzae* strain PXO524	CP013677.1
	Xanthomonas oryzae pv. *oryzae* strain PXO563	CP013678.1
	Xanthomonas oryzae pv. *oryzae* strain PXO602	CP013679.1
	Xanthomonas oryzae pv. *oryzae* strain SK2-3	CP019515.1
	Xanthomonas oryzae pv. *oryzae* strain ScYc-b	CP018087.1
	Xanthomonas oryzae pv. *oryzae* strain ScYc-b	CP031469.1
	Xanthomonas oryzae pv. *oryzae* strain T19	CP033171.1

	Xanthomonas oryzae pv. *oryzae* strain PXO364	CP033191.1
	Xanthomonas oryzae pv. *oryzae* strain PXO404	CP033190.1
	Xanthomonas oryzae pv. *oryzae* strain PXO421	CP033189.1
	Xanthomonas oryzae pv. *oryzae* strain PXO421	CP033188.1
	Xanthomonas citri pv. *malvacearum* strain AR81009	CP023155.1
	Xanthomonas hortorum pv. *gardneri* strain ICMP 7383	CP018731.1
	Xanthomonas citri subsp. *citri* strain mf20	CP009007.1
	Xanthomonas cucurbitae strain ATCC 23378	CP033326.1
	Xanthomonas cynarae strain CFBP 2044	LR828251.1
	Xanthomonas euroxanthea CPBF 424	LR994544.1
	Xanthomonas euvesicatoria strain LMG930	CP018467.1
	Xanthomonas fragariae isolate Fap21	CP016830.1
	Xanthomonas fragariae isolate Fap29	CP016833.1
	Xanthomonas fragariae strain NBC2815	LT853880.1
	Xanthomonas fragariae strain PD885	LT853882.1
	Xanthomonas fragariae strain PD5205	LT853885.1
	Xanthomonas fuscans subsp. *fragariae* strain 1566	CP012002.1
	Xanthomonas fuscans subsp. *fragariae* strain FDC 1559	CP011160.1
	Xanthomonas fuscans subsp. *fragariae* strain FDC 1561	CP011250.1
	Xanthomonas fuscans subsp. *fragariae* strain FDC 1609	CP011163.1
	Xanthomonas fuscans subsp. *fuscans* str. 4834-R	FO681494.1
	Xanthomonas gardneri strain CFBP 8129	LR828253.1
	Xanthomonas hortorum pv. *taraxaci* strain NCPPB 940	LR828264.1
