# Supplementary material for: XAC4296 Is a Multifunctional and Exclusive Xanthomonadaceae Gene Containing a Fusion of Lytic Transglycosylase and Epimerase Domains
Source: Microorganisms. 2022 May 11;10(5):1008. doi: 10.3390/microorganisms10051008 (PMC9143381; doi:10.3390/microorganisms10051008)
Supplement: Supplementary file 1 [file microorganisms-10-01008-s001.zip › Table S2.pdf]

Table S2. List of strains and plasmids used in this study.

**Bacterial strains and plasmids**	**Relevant features**	**Reference**
Xanthomonas citri; X. citri	*Xanthomonas citri* subsp. *citri*, wild type strain, citrus pathogen	(Da Silva et al., 2002)
Δ4296	*X. citri* 4296 deletion mutant of *X. citri*	This work
X. citri-p	*X. citri* harboring pMAJIIc	
Δ4296c	Complemented strain. Δ4296 harboring pMAJIIc-4296; Amp^r, Kan^r	This work
E. coli DH10B	Δ (*mcrA*, *mcrBC*, *mrr*, A1 and hsdRMS), pir+, *lacZ* for screening	Invitrogen
pNPTS138	Suicide vector, LacZ, Km^r	Oxford University
pMAJIIc	mCherry expression vector; Ap^r; Neo^r/Km^r; araC-p*ara*; amy106-912; polylinker; integrative vector in *X. citri*	(Pena et al., 2020)
pMAJIIc-4296	Recombinant vector	This work
pNPTS138-Δ4296	Suicide vector, LacZ, Km^r	This work
