# Supplementary material for: XAC4296 Is a Multifunctional and Exclusive Xanthomonadaceae Gene Containing a Fusion of Lytic Transglycosylase and Epimerase Domains
Source: Microorganisms. 2022 May 11;10(5):1008. doi: 10.3390/microorganisms10051008 (PMC9143381; doi:10.3390/microorganisms10051008)
Supplement: Supplementary file 1 [file microorganisms-10-01008-s001.zip › Table S3.pdf]

Table S3. Oligonucleotides utilized in this study.

Oligonucleotide name	Sequence (5'-3')	Restriction site	Purpose
**A (F)****	GCCTTGGCCTGGGCTAGCCTG	*Nhe*I**	Site-directed mutagenesis
**B (R)***	TCTGCCCACGGAAGGTGCCCGTAGGGCG	----	Site-directed mutagenesis
**C (R)***	CACCGCATGACGTCTGCCCACGGAAGGTGC	----	Site-directed mutagenesis
**D (F)****	GGCAAAGCTTGGGTGCTTCTGGC	*Hind*III**	Site-directed mutagenesis
**S (R) ***	CACGAGGCTTCGCACGCACC	----	Sequencing
**pMAJIIc (F) ****	ATCGCTAGCATGACGCTGTCCCGGGGC	*Nhe*I**	Complementation/mCherry fusion
**pMAJIIc (R) ****	GTCACTCGAGCTCGACGCTGATGGTCTGGGT	*Xho*I	mCherry fusion
**R4296**	CACGAGGCTTCGCACGCACC	-	Sequencing
**F4296**	GGCAAAGCTTGGGTGCTTCTGGC	-	Sequencing

*F, Forward; R, Reverse. Oligonucleotides synthesized by SIGMA-ALDRICH BRASIL.

** Underlined regions represent added restriction sites;

B/C and R1/F2 oligonucleotides blue sequences are complementary to sequences in red of its pairing primer.
