# Supplementary material for: XAC4296 Is a Multifunctional and Exclusive Xanthomonadaceae Gene Containing a Fusion of Lytic Transglycosylase and Epimerase Domains
Source: Microorganisms. 2022 May 11;10(5):1008. doi: 10.3390/microorganisms10051008 (PMC9143381; doi:10.3390/microorganisms10051008)
Supplement: Supplementary file 1 [file microorganisms-10-01008-s001.zip › Table S4.pdf]

Table S4. Live-dead quantification for XccA constructions on rich medium with ampicillin 20 µg/mL.

	**Live cells**	**Dead cells**	**%**
X. citri	300[a]	30[a]	10[a]
**Δ4296**	300[a]	37[a]	12,3[a]

Total n= 300 cells measured. Data correspond to the average cell death — same letters meaning no significant difference according to *Tukey test*- 0.05.
