# Supplementary figures and images for: XAC4296 Is a Multifunctional and Exclusive Xanthomonadaceae Gene Containing a Fusion of Lytic Transglycosylase and Epimerase Domains
Source: Microorganisms. 2022 May 11;10(5):1008. doi: 10.3390/microorganisms10051008 (PMC9143381; doi:10.3390/microorganisms10051008)

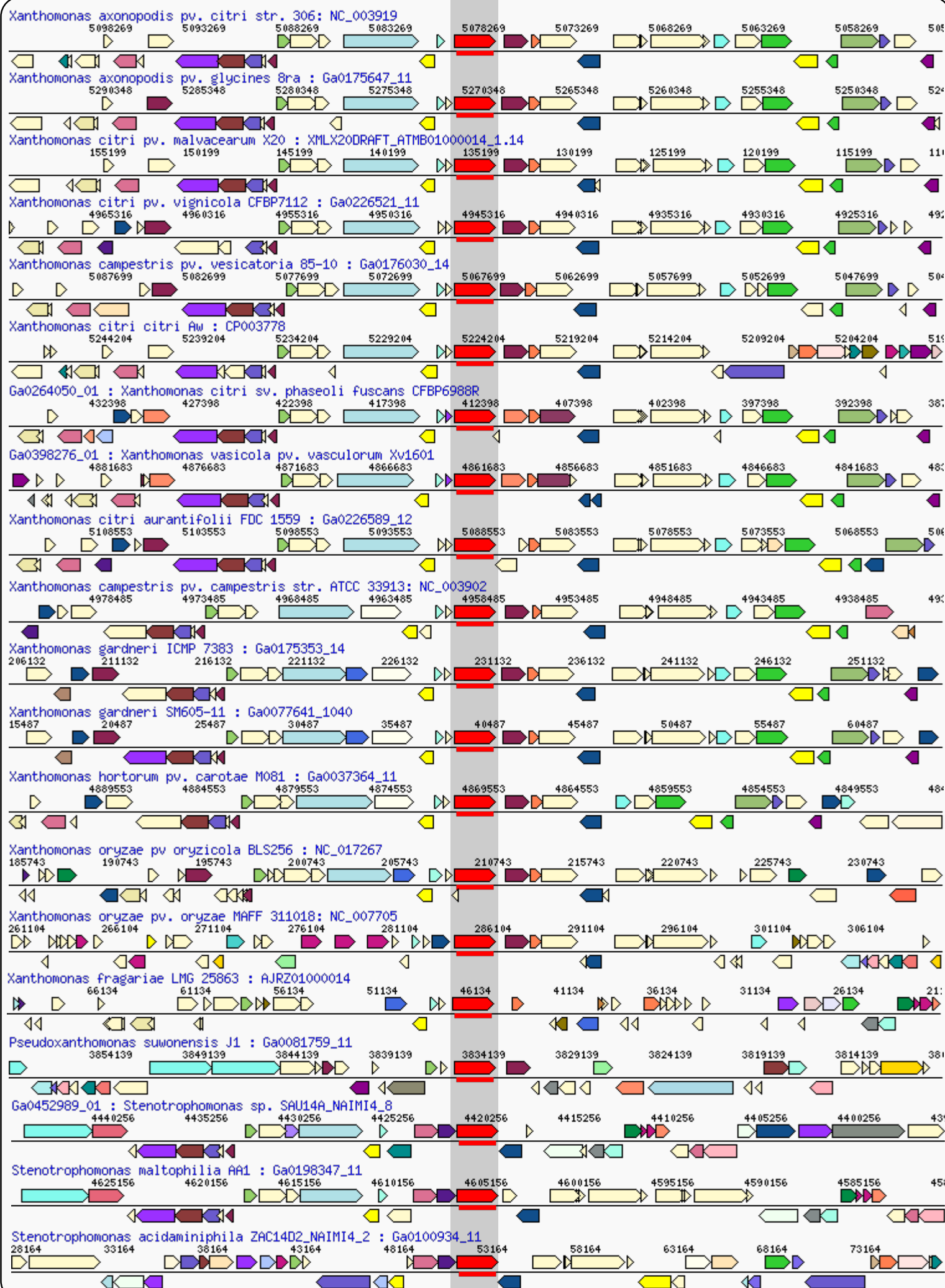

Supplement: Supplementary file 1 [file microorganisms-10-01008-s001.zip › Figure S1.pdf]

M

1

2

3

4

3000

2000

1500

500

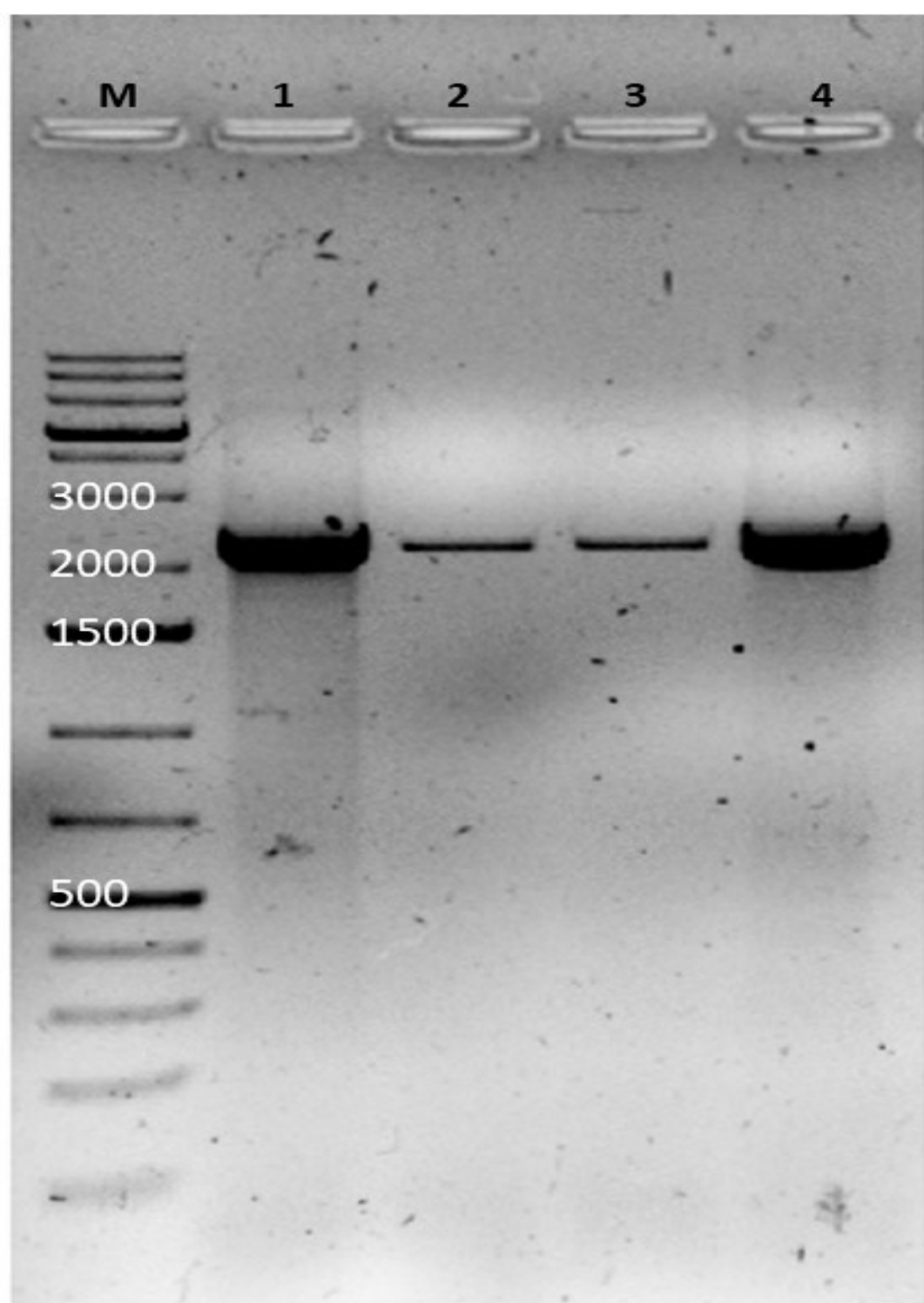

Supplement: Supplementary file 1 [file microorganisms-10-01008-s001.zip › Figure S3.pdf]

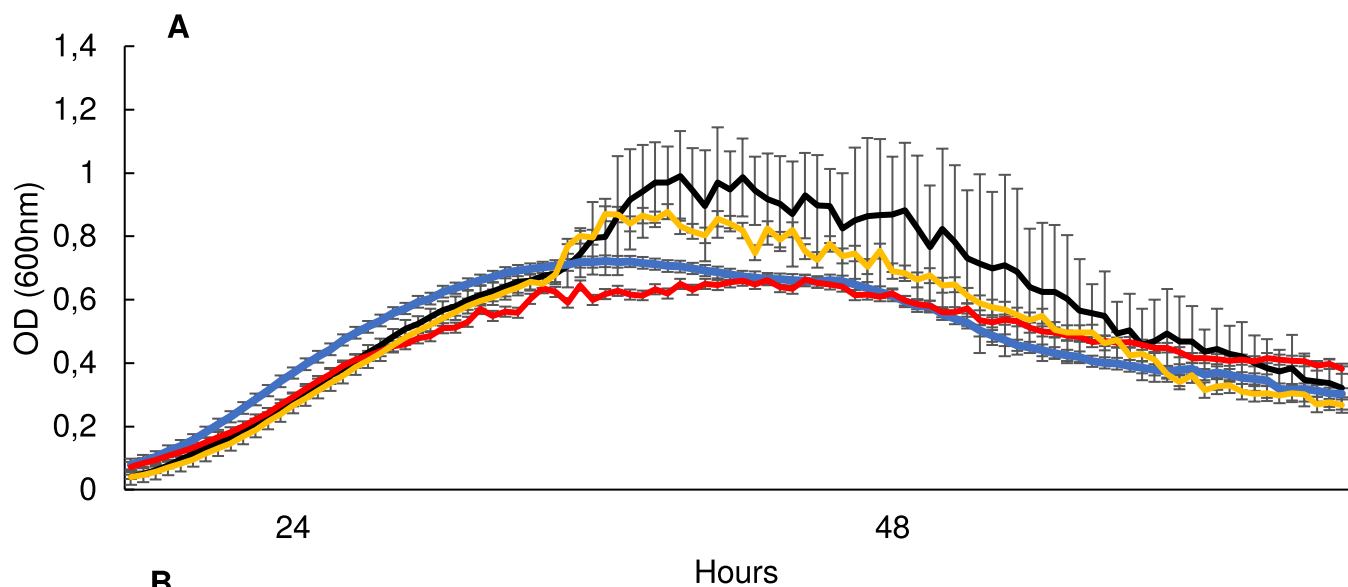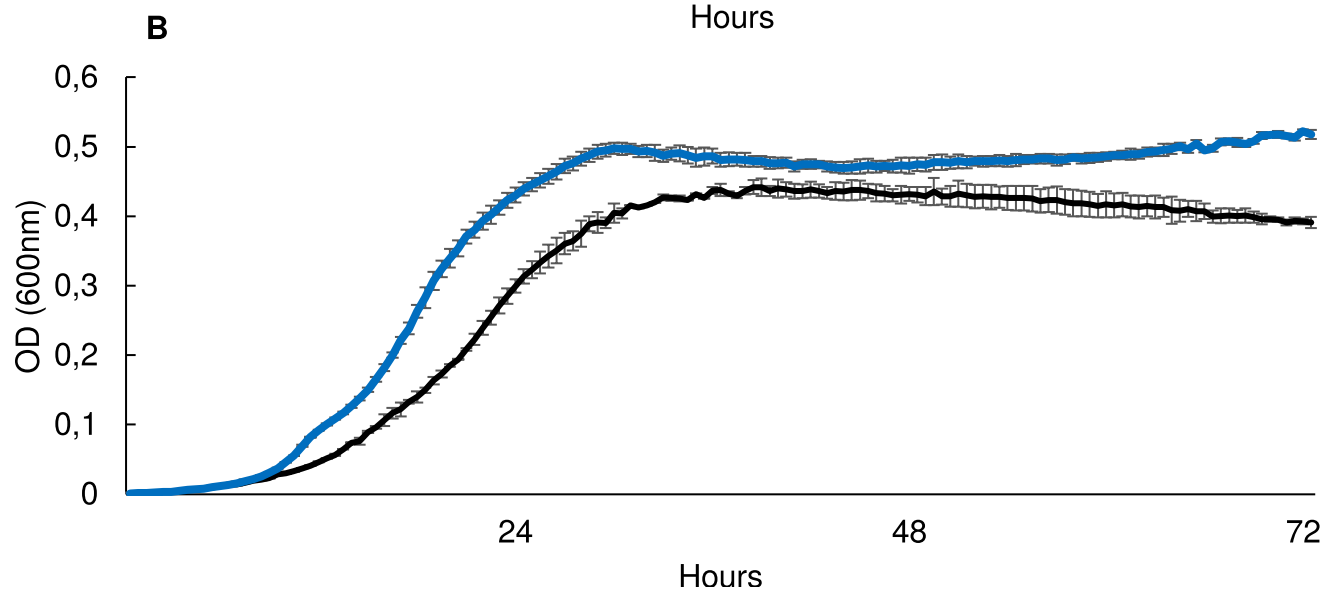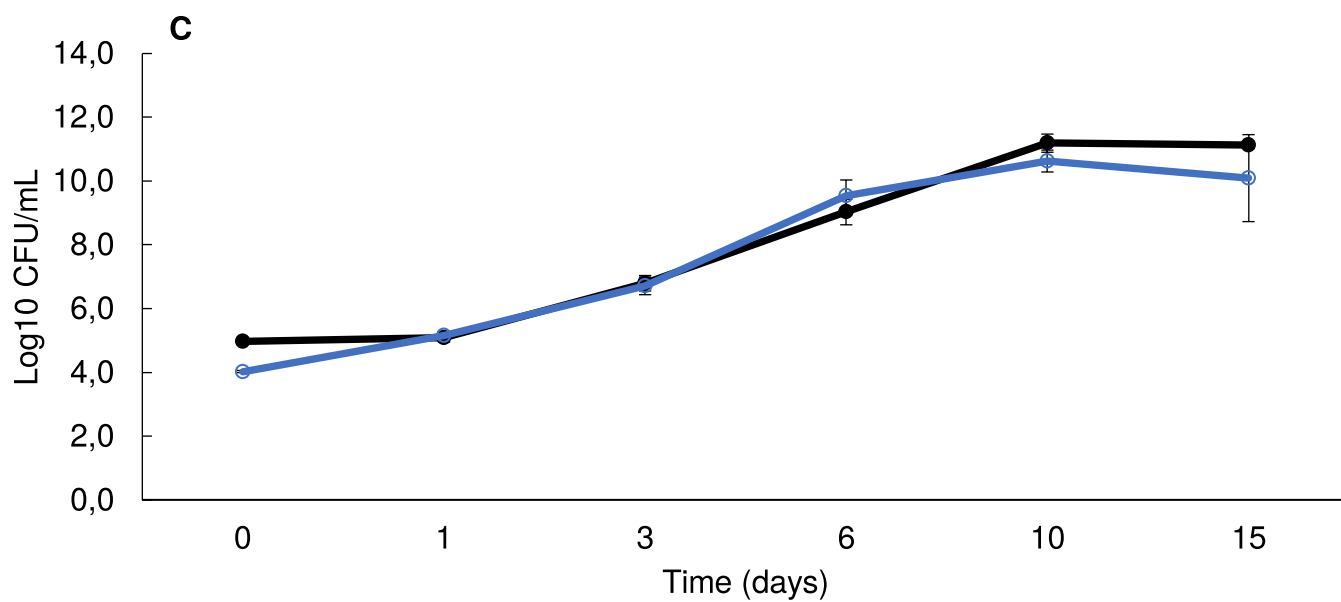

— *X. citri* —  $\Delta 4296$  —  $\Delta 4296c$  — *X. citri p*

Supplement: Supplementary file 1 [file microorganisms-10-01008-s001.zip › Figure S5.pdf]

Phase contrast

DAPI

IP

Overlay

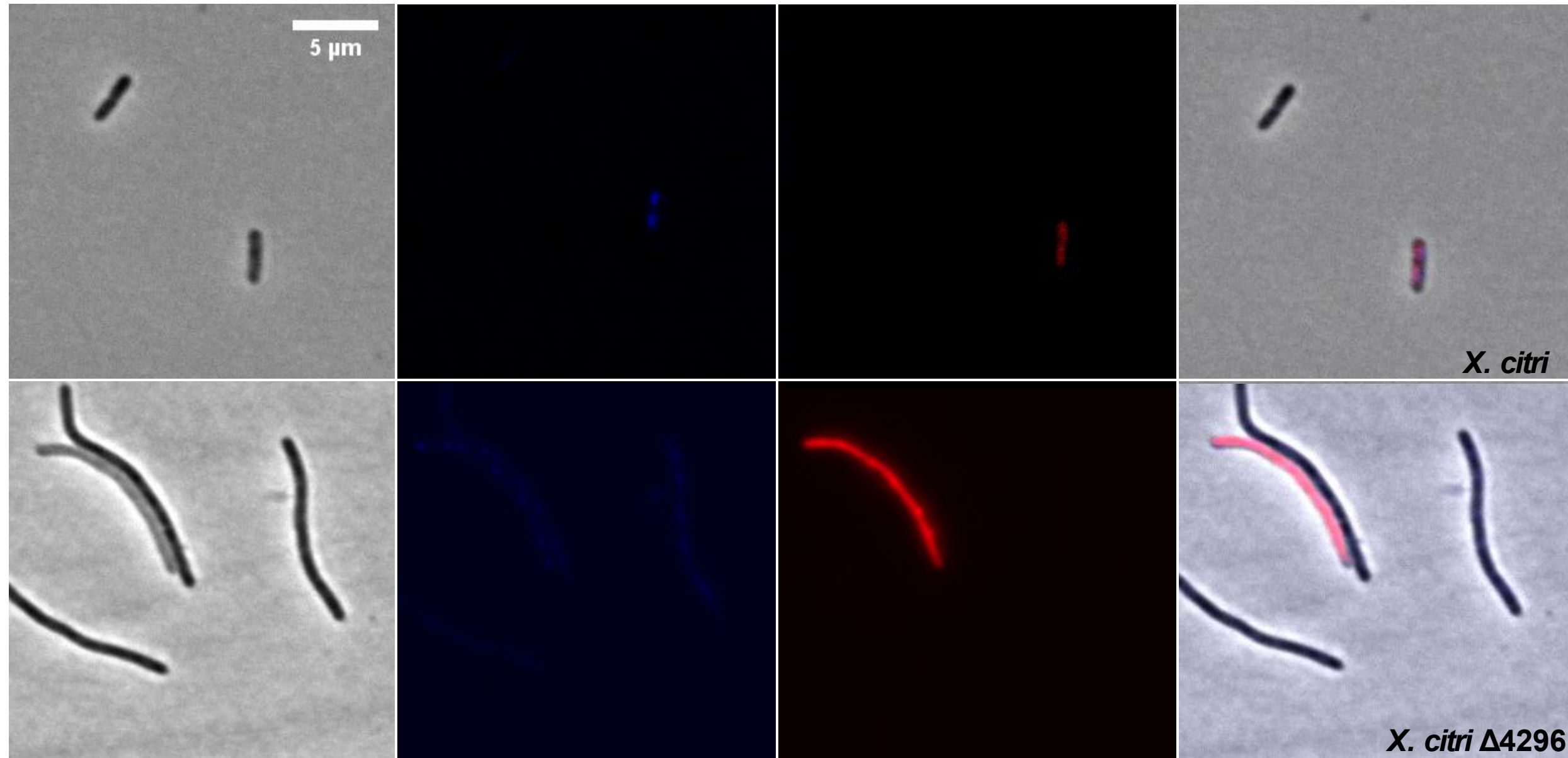

Supplement: Supplementary file 1 [file microorganisms-10-01008-s001.zip › Figure S6.pdf]

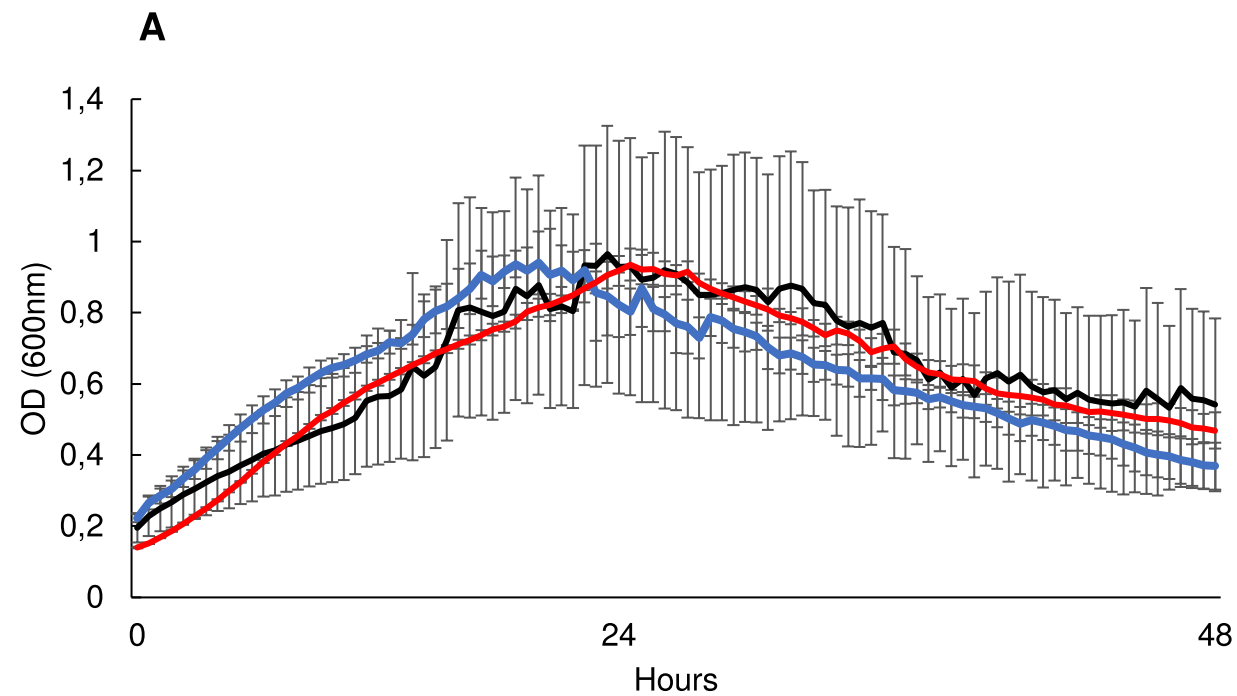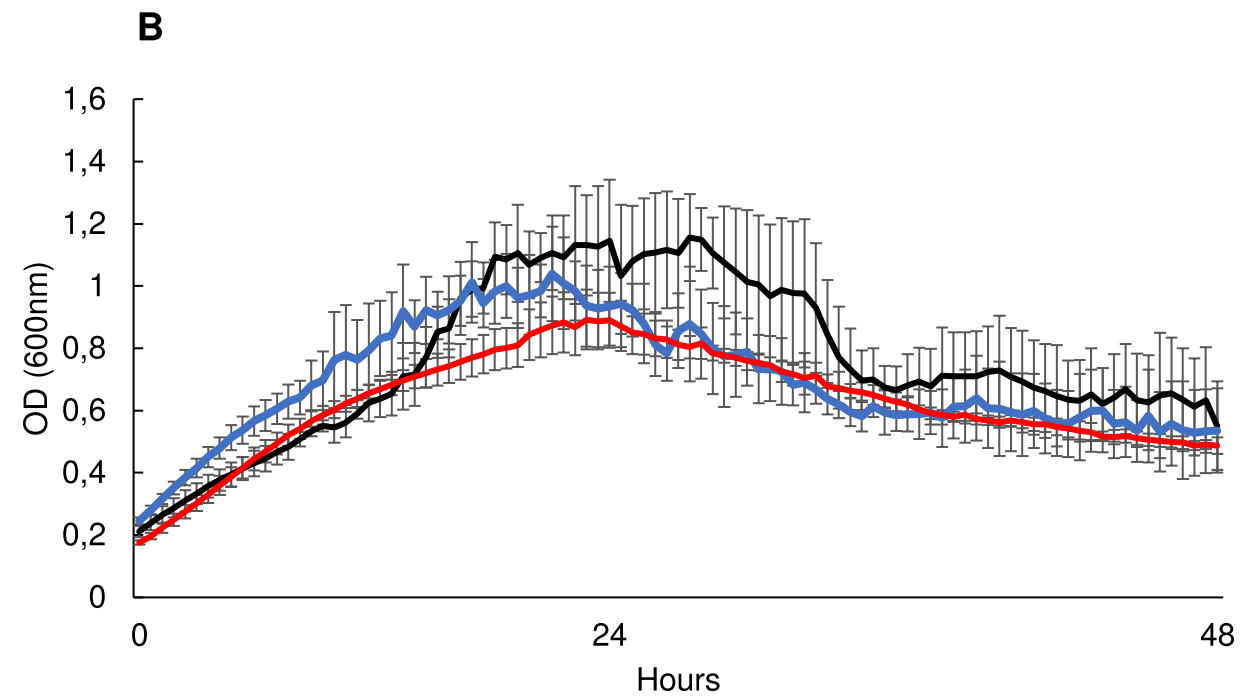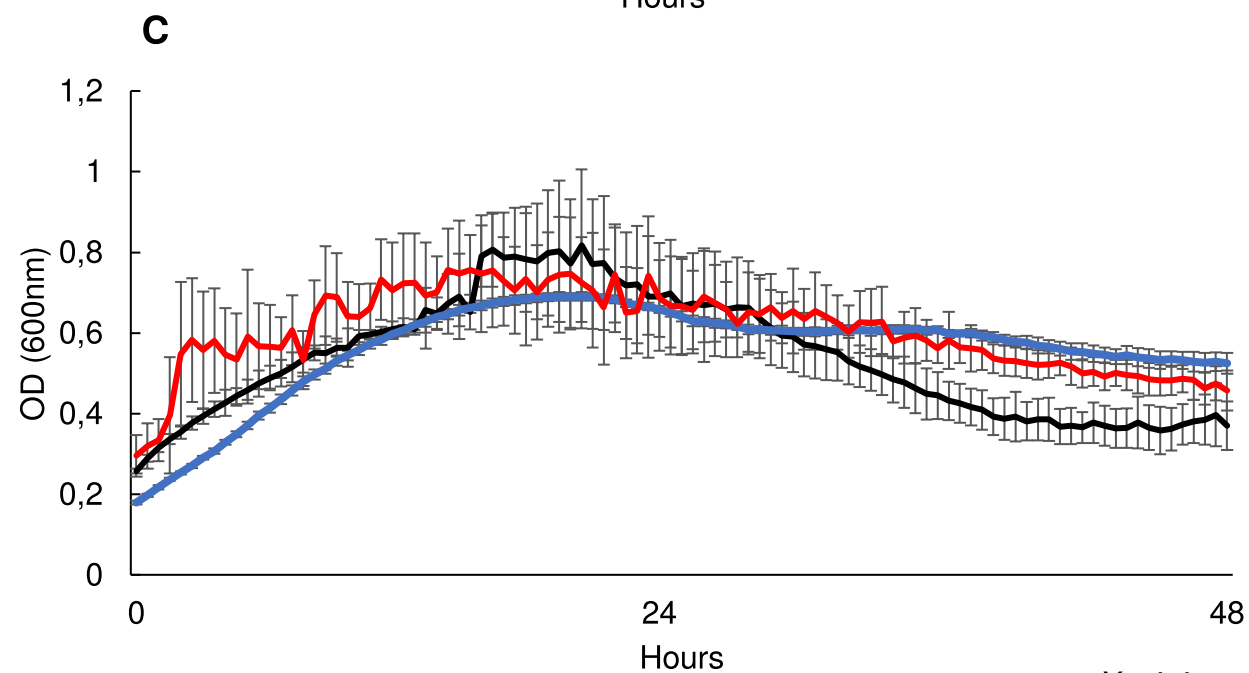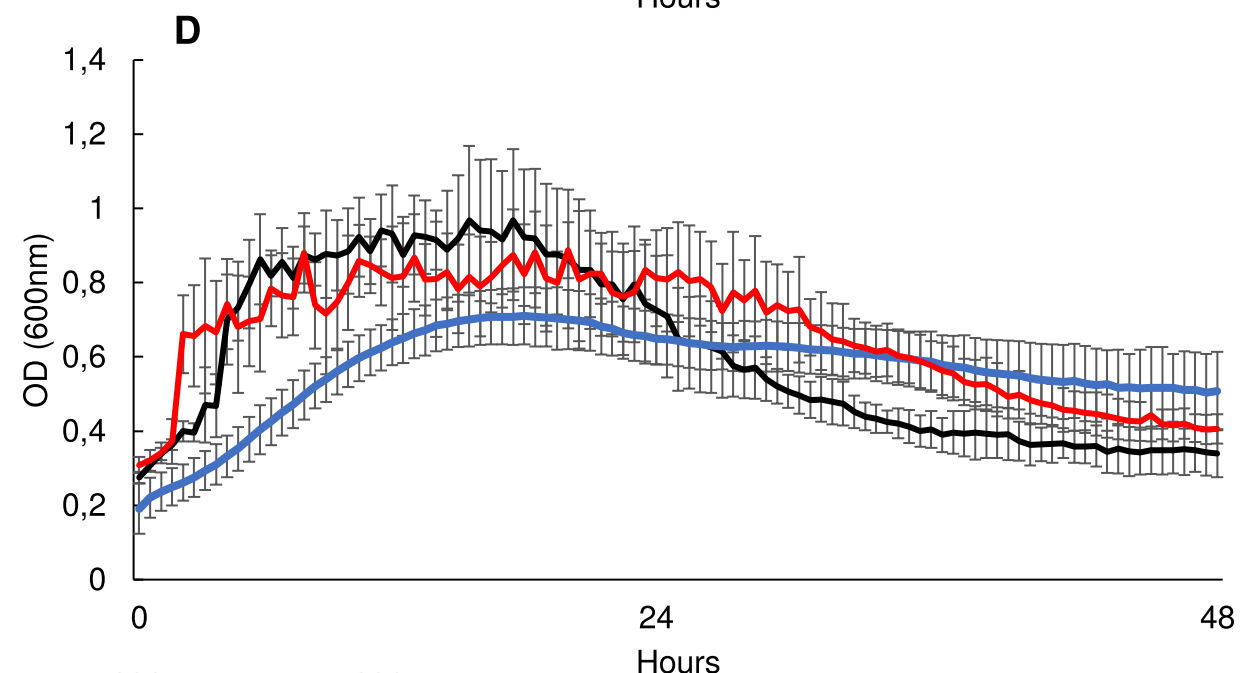

— *X. citri*

—  $\Delta 4296$

—  $\Delta 4296c$

Supplement: Supplementary file 1 [file microorganisms-10-01008-s001.zip › Figure S7.pdf]
